# Glutamate (mGluR-5) gene expression in brain regions of streptozotocin induced diabetic rats as a function of age: role in regulation of calcium release from the pancreatic islets *in vitro*

**DOI:** 10.1186/1423-0127-16-99

**Published:** 2009-11-10

**Authors:** Savitha Balakrishnan, Peeyush Kumar T, CS Paulose

**Affiliations:** 1 Molecular Neurobiology and Cell Biology Unit, Centre for Neuroscience, Department of Biotechnology, Cochin University of Science and Technology, Cochin-682 022, Kerala, India

## Abstract

Metabotrophic glutamate receptors (mGluRs) modulate cellular activities involved in the processes of differentiation and degeneration. In this study, we have analysed the expression pattern of group-I metabotropic glutamate receptor (mGlu-5) in cerebral cortex, corpus striatum, brainstem and hippocampus of streptozotocin induced and insulin treated diabetic rats (D+I) as a function of age. Also, the functional role of glutamate receptors in intra cellular calcium release from the pancreatic islets was studied *in vitro*. The gene expression studies showed that mGlu-5 mRNA in the cerebral cortex increased siginficantly in 7 weeks old diabetic rats whereas decreased expression was observed in brainstem, corpus striatum and hippocampus when compared to control. 90 weeks old diabetic rats showed decreased expression in cerebral cortex, corpus striatum and hippocampus whereas in brainstem the expression increased significantly compared to their respective controls. In 7 weeks old D+I group, mGlu-5 mRNA expression was significantly decreased in cerebral cortex and corpus striatum whereas the expression increased significantly in brainstem and hippocampus. 90 weeks old D+I group showed an increased expression in cerebral cortex, while it was decreased significantly in corpus striatum, brainstem and hippocampus compared to their respective controls. *In vitro *studies showed that glutamate at lower concentration (10^-7 ^M) stimulated calcium release from the pancreatic islets. Our results suggest that mGlu-5 receptors have differential expression in brain regions of diabetes and D+I groups as a function of age. This will have clinical significance in management of degeneration in brain function and memory enhancement through glutamate receptors. Also, the regulatory role of glutamate receptors in calcium release has immense therapeutic application in insulin secretion and function.

## Introduction

Glutamate is the major excitatory neurotransmitter in the central nervous system and exerts its action through ionotropic (iGluRs) and metabotropic receptors (mGluRs). mGluRs interact with iGluRs, ion channels and membrane enzymes that modulates cellular activities involved in the processes of differentiation and degeneration [1]. mGluRs have been divided into three subclasses according to the second-messenger pathways activated and their pharmacologic properties [2,3]. mGluR1 receptors are involved in the processing of somatosensory information as they are expressed in the thalamic neurons that receive direct sensory input [4]. Activation of group I receptors (mGluR1 and -5) results in an increase in intracellular calcium through a phospholipase C-inositoltriphosphate pathway [5] and inhibits potassium currents. The factors that modulate or disrupt IP3-mediated Ca^2+ ^signaling exert functional regulatory role in age related and other neurodegenerative disorders [6-8].

Diabetes mellitus is an endocrine disorder of carbohydrate metabolism resulting primarily from inadequate insulin release (Type I insulin-dependent diabetes mellitus) or insulin insensitivity coupled with inadequate compensatory insulin release (Type II non-insulin-dependent diabetes mellitus). Diabetes is associated with peripheral as well as central nervous system neuropathy [9,10]. Age related changes in the capacity of β-cell for proliferation affect the insulin production and contribute to a decrease in glucose tolerance with advance in age [11]. The excitatory amino acids, glutamate are pivotal elements in the hypothalamic circuitry involved in the control of pituitary function. Our previous studies reported an enhanced glutamate dehydrogenase activity during diabetes [12,13] and its regulation on brain glutamate toxicity [14]. Recent evidence suggests that metabotropic glutamate receptors are involved in the regulation of hormone secretion in the endocrine pancreas. The endogenous activation of group-I metabotropic glutamate receptors (mGlu5) are reported to be important for an optimal insulin response to glucose [15]. The functional and biochemical studies show that long-term exposure to hyperglycaemia in STZ-induced diabetic rats is associated with glutamate receptor abnormalities [16,17]. Increased glutamate content is reported to cause neuronal degeneration [18-20]. Glutamate which causes excitotoxic neuronal damage, increases calcium influx through N-methyl-D-aspartate receptors in post synaptic neurons, leading to phospholipase A_2 _mediated arachidonic acid release and neuronal injury by inhibiting the sodium ion channels [21]. mGluRs modulate several G-protein-related signal transduction pathways including intracellular calcium (iCa^2+^) that control neuronal development [22]. The synaptic activation of presynaptic Group I mGluRs increase intracellular Ca^2+ ^and an enhancement of spontaneous transmitter release [23]. Also, the role of group I and II (mGluRs) in mediating Ca^2+ ^oscillations was reported in astrocytes *in situ *[24]. In the present study, we have investigated the gene expression of group I glutamate receptors (mGluR-5), in different brain regions of streptozotocin induced diabetic rats as a function of age. Also, the functional role of glutamate receptors in intracellular calcium release from pancreatic islets was studied *in vitro*.

Glutamate is essential for synaptic communication in the CNS, but inadequate regulation of extracellular glutamate and glutamate receptor agonists can cause toxicity in the nervous system [25-27] leading to neurodegenerative disorders. Acute or chronic diabetes leads to neurological dysfunction and injury. Group I mGluRs are positively coupled to phosphoinositide hydrolysis and the mobilization of intracellular Calcium leading to excitotoxic cell death. Metabotropic glutamate (mGlu) regulates synaptic glutamate release both in vitro [28] rat brain slices [29] and in vivo [30]. The role of mGluR5 receptors in diabetes brain damage is not reported before. Our present studies on mGluR5 receptor gene expressions will definitely enlighten the involvement of glutamate in diabetes.

## Materials and methods

### Animals

Wistar weanling rats of 7-9 weeks old and 90-100 weeks old purchased from Amrita Institute of Medical Sciences, Cochin and Kerala Agricultural University, Mannuthy were used for all experiments. They were housed in separate cages under 12 hours light and 12 hours dark periods and were maintained on standard food pellets and water ad libitum. All animal care and procedures were in accordance with the CPCSEA and National Institute of Health guidelines.

### Diabetes Induction

Animals were divided into the following groups as i) Control ii) Diabetic iii) Insulin treated diabetics (D+I) of 7 weeks old and 90 weeks old rats. Each group consisted of 4-6 animals. Diabetes was induced by a single intrafemoral dose [50-60 mg/kg body weight) of streptozotocin (Sigma chemicals Co., St. Louis, MO, U.S.A.) freshly prepared in citrate buffer, pH 4.5 under anesthesia [31-33]. The control rats were given the citrate buffer injection. The insulin treated diabetic group received a daily dose (1 Unit/kg body weight) of Lente and Plain insulin (Abbott India). The dose was increased daily according to the blood glucose level [34]. Blood glucose was estimated by glucose-oxidase peroxidase method using Glucose estimation kit (Merck).

### Sacrifice and tissue preparation

The animals were then sacrificed on 15^th ^day by decapitation. The brain regions were dissected out quickly over ice according to the procedure of Glowinski and Iversen, [35]. The tissues were stored at -70°C until assay.

### Analysis of gene expression by Real-Time PCR

Total RNA was isolated from the brain regions of control and experimental rats using Tri reagent. RNA was reverse transcribed using ABI PRISM cDNA Archive kit. 20 μl of the reaction mixture contained 0.2 μg total RNA, 10× RT buffer, 25× dNTP mixture, 10× Random primers, MultiScribe RT (50 U/μl) and RNase free water. The reactions were carried out at 25°C for 10 minutes and 37°C for 2 hours using an Eppendorf Personal Cycler. Real-Time PCR assays were performed using specific primer and fluorescently labeled Taq probe in an ABI 7300 Real-Time PCR instrument (Applied Biosystems). The TaqMan reaction mixture of 20 μl contained 25 ng of total RNA-derived cDNAs, 200 nM each of the forward primer, reverse primer and TaqMan probe for mGlu-5 gene, endogenous control, β-actin and 12.5 μl of TaqMan 2× Universal PCR Mastermix. The thermocycling profile conditions used were: 50°C - 2 minutes - Activation; 95°C - 10 minutes - Initial Denaturation; followed by 40 cycles of 95°C - 15 seconds - Denaturation and 60°C-1 minute - Annealing. The ΔΔCT method of relative quantification was used to determine the fold change in expression. This was done by first normalizing the resulting threshold cycle (CT) values of the target mRNAs to the CT values of the internal control β-actin in the same samples (ΔCT = CT_Target _- CT_β-actin_). It was further normalized with the control (ΔΔCT = ΔCT - CT_Control_). The fold change in expression was then obtained as (2^-ΔΔCT^) and expressed as log 2^-ΔΔCT^.

### Primer Sequence

Beta actin: **TCA CCC ACA CTG TGC CCC ATC TAC GA**

mGlu-5 receptor: **TTCTGGGCAGTGATGGCTGGGCCGA**

### Isolation of pancreatic islets

Pancreatic islets were isolated from 20 weeks old adult Wistar rats by standard collagenase digestion procedures using aseptic techniques [36]. The islets were isolated in HEPES-buffered sodium free Hanks Balanced Salt Solution (HBSS) [37] with the following composition: 137 mM Choline chloride, 5.4 mM KCl, 1.8 mM CaCl_2_, 0.8 mM MgSO_4_, 1 mM KH_2_PO_4_, 14.3 mM KHCO_3 _and 10 mM HEPES. Autoclaved triple distilled water was used in the preparation of the buffer. The pancreas from both young and old rats was aseptically dissected out into a sterile petridish containing ice cold HBSS and excess fat and blood vessels were removed. The pancreas was cut into small pieces and transferred to a sterile glass vial containing 2 ml collagenase type XI solution (1.5 mg/ml in HBSS, pH 7.4). The collagenase digestion was carried out for 15 minutes at 37°C in an environmental shaker with vigorous shaking (300 rpm/minute). The tissue digest was filtered through 500 μm nylon screen and the filtrate was washed with three successive centrifugations and resuspensions in cold HBSS. The pancreatic islet preparation having a viability of >90% as assessed by Trypan Blue exclusion which was chosen for cell culture and other experiments.

### Calcium imaging

Pancreatic islets were prepared from 20 weeks old adult Wistar rats by collagenase digestion method as mentioned earlier. The isolated islets were incubated for 4 hours at room temperature in 1 ml of RPMI medium containing 5 μM of Ca^2+ ^fluorescent dye, fluo 4-AM (Molecular Probes, Eugene, OR), to monitor the changes in the intracellular Ca^2+^. After incubation cells were washed twice in indicator free RPMI medium to remove excess dye that was non-specifically associated with the cell surface, and then incubated for further 30 minutes to allow complete de esterification of intra cellular AM esters. The 35 mm plates, containing pancreatic islet cells were placed on the stage of a Leica TCS SP5 laser scanning confocal microscope equipped with a HC PL FLUOTAR 20.0 × 0.50 dry objective (NA 0.5). Fluo 4-AM was excited with 514 nm laser lines from an argon laser, with laser intensity set at 38% of available power. For visualization of Fluo 4-AM, the emission window was set at 508.4 nm - 571.5 nm. The images were continuously acquired before and after addition of both higher (10^-4 ^M) and lower concentration (10^-7 ^M) of glutamate, at time intervals of 1.318 seconds, for a total of 600 seconds. Time series experiments were performed collecting 512 × 512 pixel images at 400 Hz. Fluorescence intensity was analysed using the quantitation mode in LAS-AF software from Leica Microsystems, Germany. A region of interest (ROI) was drawn within a field of view. For each ROI, the pixel intensity was calculated for each image in the 600 seconds sequence to analyse the intracellular Ca^2+ ^release from the pancreatic islet cells in experimental conditions.

### Statistics

Statistical evaluations were performed by Student's t-Test and ANOVA by using Graphpad InStat (Ver.2.04a, San Diego, USA) computer program. Relative Quantification Software was used for analyzing Real-Time PCR results.

## Results

The body weight was significantly decreased (p < 0.001) in 7 weeks old and 90 weeks old diabetic rat groups when compared to their respective controls. After insulin treatment for 14 days, the body weight was reversed to near the initial level in both groups (Table 1, 2). Blood glucose level of all rats before streptozotocin administration was within the normal range. Streptozotocin administration led to a significant increase (p < 0.001) in blood glucose level of 7 weeks old and 90 weeks old diabetic rat groups when compared to their respective controls. In both groups, insulin treatment significantly reduce (p < 0.001) the increased blood glucose level to near the control value when compared to diabetic group (Table 3, 4).

**Table 1:** Body weight (g) of Experimental rats (7 weeks old)

Animal status	Initial	7^th ^day	14^th ^day
**Control**	110.0 ± 10.0	120.5 ± 12.0	133.3 ± 14.5
**Diabetic**	120.0 ± 5.7	86.6 ± 4.6	75.7 ± 3.6*** ^ϕϕϕ^
**Insulin treated Diabetic**	116.6 ± 3.4	98.2 ± 2.0	106.6 ± 5.3^¶¶¶^

Values are mean ± S.E.M of 4-6 rats in each group.

***p < 0.001 when compared with control, ^ϕϕϕ^p < 0.001 when compared with initial weight, ^¶¶¶^p < 0.001 when compared with diabetic group.

**Table 2:** Body weight (g) of Experimental rats (90 weeks old)

Animal status	Initial	7^th ^day	14^th ^day
**Control**	320.0 ± 12.0	325.0 ± 14.3	330.0 ± 15.0
**Diabetic**	360.0 ± 16.5	280.2 ± 3.3	250.7 ± 2.3*** ^ϕϕϕ^
**Insulin treated Diabetic**	350.0 ± 13.3	300.6 ± 5.0	310.0 ± 5.8^¶¶¶^

Values are mean ± S.E.M of 4-6 rats in each group.

***p < 0.001 when compared with control, ^ϕϕϕ^p < 0.001 when compared with initial weight, ^¶¶¶^p < 0.001 when compared with diabetic group.

**Table 3:** Blood glucose (mg/dL) level in Experimental rats (7 weeks old)

Animal status	0 day (Before STZ injection)	3^rd ^day (Initial)	6^th ^day	10^th ^day	14^th ^day (Final)
**Control**	108.6 ± 1.7	113.1 ± 0.8	120.5 ± 1.5	123.7 ± 0.7	127.5 ± 0.9
**Diabetic**	105.9 ± 0.7	256.2 ± 0.4	301.2 ± 0.5	305.7 ± 0.7	306.9 ± 1.3***
**D + I**	113.5 ± 1.2	258.8 ± 0.3	298.4 ± 0.9	182.8 ± 1.3	130.0 ± 1.1^¶¶¶ ^^ϕϕϕ^

Values are mean ± S.E.M of 4-6 rats in each group.

*** P < 0.001 when compared to control, ^¶¶¶^P < 0.001 when compared to diabetic group,

^ϕϕϕ^p < 0.001 when compared with initial reading.

D + I- Insulin treated Diabetic

**Table 4:** Blood glucose (mg/dL) level in Experimental rats (90 weeks old)

Animal status	0 day (Before STZ injection)	3^rd ^day (Initial)	6^th ^day	10^th ^day	14^th ^day (Final)
**Control**	110.1 ± 1.5	120.2 ± 1.7	122.5 ± 0.6	127.9 ± 2.1	128.3 ± 1.21
**Diabetic**	115.7 ± 1.2	248.9 ± 1.5	260.5 ± 0.7	265.7 ± 0.4	267.9 ± 1.2***
**D + I**	119.2 ± 0.7	254.8 ± 0.6	262.4 ± 0.9	193.8 ± 1.2	164.6 ± 0.9^¶¶¶ ^^ϕϕϕ^

Values are mean ± S.E.M of 4-6 rats in each group.

***P < 0.001 when compared to control, ^¶¶¶^P < 0.001 when compared to diabetic group,

^ϕϕϕ^p < 0.001 when compared with initial reading.

D + I- Insulin treated Diabetic

**Figure 1:**
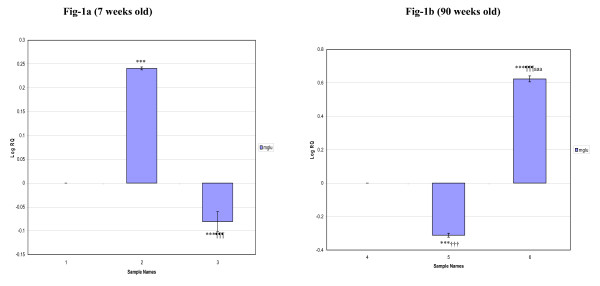
**Real-Time PCR amplification of Glutamate receptor (mGlu-5) mRNA from the Cerebral cortex of Control, Diabetic and Insulin treated Diabetic Rats**. **Sample Names**. 1. 7 weeks Control, 2. 7 weeks Diabetic, 3. 7 weeks Insulin treated Diabetic. 4. 90 weeks Control, 5. 90 weeks Diabetic, 6. 90 weeks Insulin treated Diabetic. Real Time PCR analysis was done using mGlu-5 specific primer and fluorescently labeled Taq probe. The TaqMan reaction mixture of 20 μl contained 25 ng of total RNA-derived cDNAs, 200 nM each of the forward primer, reverse primer and TaqMan probe for glutamergic- (mGlu-5) gene, endogenous control, β-actin and 12.5 μl of TaqMan 2× Universal PCR Mastermix (Applied Biosystems). All reactions were performed in duplicates. The ΔΔCT method of relative quantification was used to determine the fold change in expression. This was done by first normalizing the resulting threshold cycle (CT) values of the target mRNAs to the CT values of the internal control β-actin in the same samples (ΔCT = CT_Target _- CT_β-actin_). It was further normalized with the control (ΔΔCT = ΔCT - CT_Control_). The fold change in expression was then obtained as (2^-ΔΔCT^) and expressed as log 2^-ΔΔCT^. Values are Mean ± S.D of 4-6 separate experiments. ***p < 0.001 when compared to control, ^¶¶¶ ^p < 0.001 when compared to diabetic, ^††† ^p < 0.001 when compared to 7 weeks old diabetic, ^aaa ^p < 0.001 when compared to 7 weeks old insulin treated diabetic.

The gene expression studies using Real-Time PCR showed that the glutamate receptor (mGlu-5) mRNA in the cerebral cortex was increased siginficantly (p < 0.001) in 7 weeks old diabetic rats whereas decreased expression (p < 0.001) was observed in D+I group compared to control. 90 weeks old diabetic rats showed decreased expression (p < 0.001) while D+I group showed an increased expression (p < 0.001) compared to control. Also, the expression was decreased significantly in 90 weeks old diabetic rats (p < 0.001) whereas an increased expression pattern (p < 0.001) was observed in D+I groups compared to 7 weeks old diabetic and D+I groups respectively (Fig 1).

**Figure 2:**
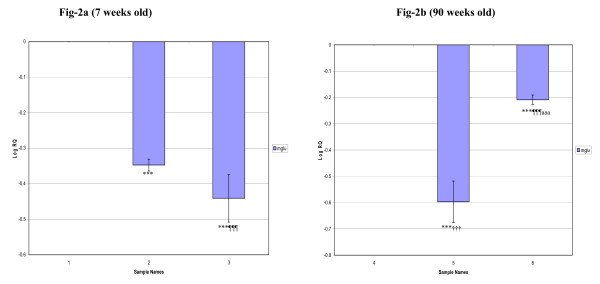
**Real-Time PCR amplification of Glutamate receptor (mGlu-5) mRNA from the Corpus striatum of Control, Diabetic and Insulin treated Diabetic Rats**. **Sample Names**. 1. 7 weeks Control, 2. 7 weeks Diabetic, 3. 7 weeks Insulin treated Diabetic. 4. 90 weeks Control, 5. 90 weeks Diabetic, 6. 90 weeks Insulin treated Diabetic. Values are Mean ± S.D of 4-6 separate experiments. ***p < 0.001 when compared to control, ^¶¶¶^p < 0.001 when compared to diabetic, ^†††^p < 0.001 when compared to 7 weeks old diabetic, ^aaa^p < 0.001 when compared to 7 weeks old insulin treated diabetic. **Other details as in the legend to Figure-1**.

**Figure 3:**
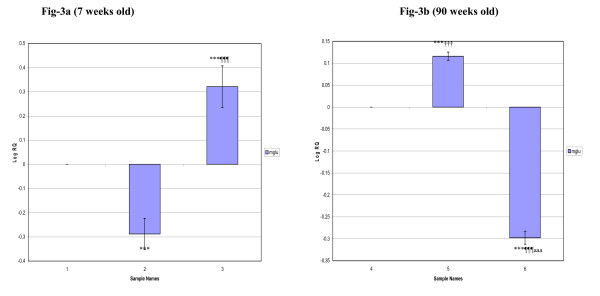
**Real-Time PCR amplification of Glutamate receptor (mGlu-5) mRNA from the Brainstem of Control, Diabetic and Insulin treated Diabetic Rats**. **Sample Names**. 1. 7 weeks Control, 2. 7 weeks Diabetic, 3. 7 weeks Insulin treated Diabetic. 4. 90 weeks Control, 5. 90 weeks Diabetic, 6. 90 weeks Insulin treated Diabetic. Values are Mean ± S.D of 4-6 separate experiments. ***p < 0.001 when compared to control, ^¶¶¶^p < 0.001 when compared to diabetic, ^†††^p < 0.001 when compared to 7 weeks old diabetic, ^aaa^p < 0.001 when compared to 7 weeks old insulin treated diabetic. **Other details as in the legend to Figure-1**.

In the corpus striatum, the gene expression decreased significantly (p < 0.001) in 7 weeks and 90 weeks old diabetic and D+I groups when compared to control. In 90 weeks old diabetic group, we observed a decreased expression (p < 0.001) whereas D+I group showed an increased expression (p < 0.001) compared to 7 weeks old diabetic and D+I groups respectively (Fig 2). In the brainstem, mGlu-5 mRNA expression was significantly decreased (p < 0.001) in 7 weeks old diabetic whereas it was significantly increased (p < 0.001) in D+I groups compared to control. 90 weeks old diabetic rats showed an increased expression while D+I group showed a decreased expression (p < 0.001) compared to control. Also, an increased expression of mGlu-5 receptor mRNA was observed in 90 weeks old diabetic (p < 0.001) whereas D+I groups showed decreased expression (p < 0.001) compared to 7 weeks old diabetic and D+I groups respectively (Fig 3).

**Figure 4:**
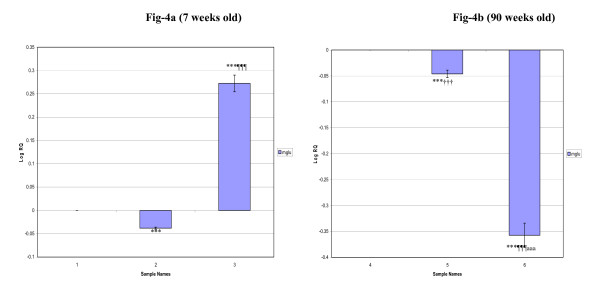
**Real-Time PCR amplification of Glutamate receptor (mGlu-5) mRNA from the Hippocampus of Control, Diabetic and Insulin treated Diabetic Rats**. **Sample Names**. 1. 7 weeks Control, 2. 7 weeks Diabetic, 3. 7 weeks Insulin treated Diabetic. 4. 90 weeks Control, 5. 90 weeks Diabetic, 6. 90 weeks Insulin treated Diabetic. Values are Mean ± S.D of 4-6 separate experiments. ***p < 0.001 when compared to control, ^¶¶¶^p < 0.001 when compared to diabetic, ^†††^p < 0.001 when compared to 7 weeks old diabetic, ^aaa^p < 0.001 when compared to 7 weeks old insulin treated diabetic. **Other details as in the legend to Figure-1**.

The hippocampus elicit an increased mGlu-5 receptor mRNA expression (p < 0.001) in 7 weeks old D+I groups whereas it was significantly decreased (p < 0.001) in 7 weeks diabetic and 90 weeks old rat groups compared to their respective controls. Also, a significant decrease in the expression was observed in 90 weeks old diabetic (p < 0.001) and D+I (p < 0.001) groups compared to 7 weeks old diabetic and D+I groups respectively (Fig 4).

**Figure 5:**
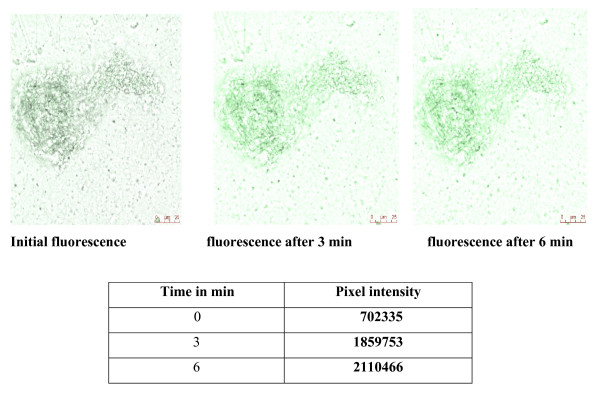
**The isolated pancreatic islets were incubated for 4 hours at room temperature in 1 ml of RPMI medium containing 5 μM of Ca^2+ ^fluorescent dye, fluo 4-AM**. The images were continuously acquired before and after addition of 10^-7 ^M of glutamate, at time intervals of 1.318 seconds, for a total of 600 seconds. Time series experiments were performed collecting 512 × 512 pixel images at 400 Hz. Fluorescence intensity was analysed using the quantitation mode in LAS-AF software from Leica Microsystems, Germany. A region of interest (ROI) was drawn within a field of view. The pixel intensity was calculated for each image in the 600 seconds sequence to analyse the intracellular Ca^2+ ^release from the pancreatic islet cells in experimental conditions.

**Figure 6:**
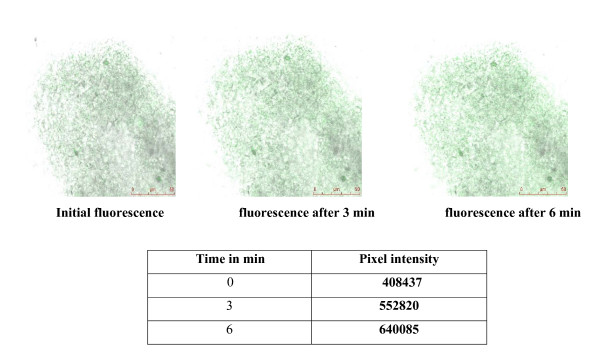
**The isolated pancreatic islets were incubated for 4 hours at room temperature in 1 ml of RPMI medium containing 5 μM of Ca^2+ ^fluorescent dye, fluo 4-AM**. The images were continuously acquired before and after addition of 10^-4 ^M of glutamate, at time intervals of 1.318 seconds, for a total of 600 seconds. Time series experiments were performed collecting 512 × 512 pixel images at 400 Hz. Fluorescence intensity was analysed using the quantitation mode in LAS-AF software from Leica Microsystems, Germany. A region of interest (ROI) was drawn within a field of view. The pixel intensity was calculated for each image in the 600 seconds sequence to analyse the intracellular Ca^2+ ^release from the pancreatic islet cells in experimental conditions.

*In vitro *studies showed that glutamate at 10^-7 ^M significantly increased Ca^2+ ^release from the pancreatic islets (Fig 5, 6).

## Discussion

Glutamate is an excitatory neurotransmitter in the mammalian central nervous system and mediates neurotransmission across most excitatory synapses. Metabotrophic glutamate receptors (mGluRs) are mainly found in pre and postsynaptic neurons in synapses of the hippocampus, cerebellum and the cerebral cortex as well as other parts of the brain and in peripheral tissues. Previous reports suggest the in adult rats mGluR-5 is expressed in many brain regions highest in striatum, hippocampus frontal cortex and relatively low in cerebellum and pons medulla [38,39]. mGluR-5 receptors expression is precisely and complexly controlled at the level of transcription and that different functions of mGlu5 during different developmental periods and in distinct regions are likely mediated by different splice variants [40].

The progression of diabetes is associated with an impaired ability of the neurons in the CNS to release neurotransmitters resulting in behavioral changes [41]. Among them, the neurotransmitter receptor NMDA showed age-related reduction of expression [42]. The cerebral cortex is the seat of our highest forms of intelligence. It plays a central role in many complex brain functions including memory, attention, perceptual awareness, thought, language, and consciousness. Glutamate triggers neuronal death when released in excessive concentrations by overexcitation of its receptors [43]. Age-related neuronal dysfunction causes a decline in cognitive function and other subtle changes within the cortex including alterations in receptors, loss of dendrites, and spines and myelin dystrophy, as well as the alterations in synaptic transmission [44]. Glutamate receptor subtypes are critical in gating the plasticity and memory formation. Also, learning and memory deficits are associated with Type I and Type II diabetes mellitus [45] and brain morphological abnormalities have been found in diabetic patients, mainly in the cortex area [46]. Studies on streptozotocin (STZ)-induced diabetic rat models have shown similar results which exhibits morphological, behavioural and electrophysiological alterations on diabetes [47-49]. Increased blood glucose and decreased body weight observed during diabetes is similar with previous reports as a result of the marked destruction of insulin secreting pancreatic islet β-cells by streptozotocin [50]. During diabetes there is a decrease in body weight as a result of altered metabolic function. Insulin treatment normalised the increased blood glucose level and decreased body weight to control values.

Abnormal expression of glutamate receptor is also involved in the development of diabetic neuropathy [51]. The brain regions - cerebral cortex and hippocampus of diabetic rats is suggested to be more vulnerable to glutamate toxicity via NMDA receptor activation. An Age-related increase in group-I mGlu receptor mRNA levels was found in thalamic nuclei, hippocampal CA3 with parallel increases in mGlu receptor protein expression [52]. Diabetes mellitus induces cognitive impairment and defects of long-term potentiation (LTP) in the hippocampus. From the gene expression studies, it is clear that in cerebral cortex, mGlu-5 mRNA increased siginficantly in 7 weeks old diabetic rats compared to control groups. Aging process affects NMDA receptors more in the intermediate hippocampus than the dorsal [53]. The dysfunction in hippocampal LTP, an electrophysiological model of synaptic plasticity thought to subserve learning and memory processes is associated with diabetic conditions [54,55]. Also, L- [(3)H]glutamate-labeled NMDA receptors were found to be down regulated in primary sensory cortical regions [56]. Brain stem is an important part of the brain in monitoring the glucose status and the regulation of feeding [57]. The dorsal motor nucleus of the vagus nerve is located in the brainstem. It is connected to the endocrine pancreas exclusively via vagal fibres and has a role in neurally mediated insulin release. Hippocampus is based on recent or declarative memory and plays important roles in long-term memory and spatial navigation. In brainstem, corpus striatum and hippocampus, we observed a decreased expression of mGlu-5 mRNA when compared to control groups. 90 weeks old diabetic rats showed decreased expression in cerebral cortex, corpus striatum and hippocampus whereas in the brainstem the expression increased significantly. Corpus striatum is best known for its role in the planning and modulation movement pathways but also involved in a variety of cognitive process involving executive function. Studies show an age-dependent reduction in the functional response of striatal group I mGlu receptors, which may be one of the factors underlying the reduced ability of aged striatum to integrate information [58]. Our results suggest that glutamate receptor alterations found in the brain regions contribute to cognitive and memory deficits during diabetes as a function of age.

Studies have shown that regulation of glutamate receptor properties can contribute to learning and memory [59]. Activation of this neurotransmitter system is also involved in neurodegeneration following a wide range of neurological insults, including ischemia, trauma and epileptic seizures [60,61]. In rodents, N-methyl-D-aspartate (NMDA) and non-NMDA receptors are two families of ionotropic receptors stimulated by glutamate that have been implicated in neurodegeneration [62]. Overactivation of these receptors can cause cell damage by increasing intracellular calcium concentration in neurons, thereby leading to the generation of free radicals and activation of proteases, phospholipases and endonucleases [63,64] as well as transcriptional activation of specific cell death programs [65].

In 7 weeks old D+I group, mGlu-5 mRNA expression was significantly decreased in cerebral cortex and corpus striatum whereas the expression increased significantly in brainstem and hippocampus. 90 weeks old D+I group showed an increased expression in cerebral cortex, while it was decreased significantly in corpus striatum, brainstem and hippocampus compared to their respective controls. Insulin is reported to regulate the reuptake of catecholamine transporters. Intracerebroventricular injection of insulin is reported to cause an increased mRNA expression of dopamine transporters [66]. Short-term insulin treatment was found to alter NMDA receptor activation [67] as well as to interact with AMPA receptor trafficking between the plasma membrane and the intracellular compartment in neuronal cell culture [68] indicating that mechanisms underlying diabetic neuropathies could be initiated in the early stages of the disease, as a consequence of abnormal glutamate receptor properties. This is relevant to the clinical situation because excessive activation of glutamate receptors is a characteristic feature of brain damage during stroke and ischaemia [69], conditions that are exacerbated by hyperglycaemic states.

Intracellular Ca^2+ ^plays a major role in the physiological responses of excitable cells and excessive accumulation of internal Ca^2+ ^is a key determinant of cell injury and death [70]. Pancreatic islet cells express receptors and transporters for L-glutamate and use L-glutamate as an intercellular signaling molecule [71]. Both α and β-cells possess functional vesicular glutamate transporters regulated by alteration in glucose concentration *via *the transcriptional mechanism [72]. Maechler and Wollheim [73] recently provided evidence that glutamate acts downstream of the mitochondria by sensitizing the Ca^2+^-mediated exocytotic process. Our studies showed that glutamate at lower concentration (10^-7 ^M) increased Ca^2+ ^release from the pancreatic islet cells of male Wistar rats *in vitro*. Also, the stimulatory effect of glutamate on Ca^2+ ^release was found to be decreased at higher concentration (10^-4 ^M). The activation of mGluR increase intracellular calcium through a phospholipase C-inositoltriphosphate pathway [74] and inhibits potassium currents. The resultant cytosolic Ca^2+ ^transients serve numerous signaling functions, including modulation of membrane excitability [75,76], synaptic plasticity [77,78], and gene expression [79]. Glutamate receptors regulate Ca^2+^-dependent secretory mechanisms in islet cells by altering the membrane potential of these cells. The ability of mGluR to increase intracellular Ca^2+ ^depends on their co-expression with voltage-gated Ca^2+ ^channels and on the ionic gradients present in the cells in which they are expressed [80]. The metabolic changes in islet cells reported an increase in cellular ATP that closes ATP-sensitive potassium channels causing depolarization of the plasma membrane potential. Subsequently, depolarization opens voltage-sensitive Ca^2+ ^channels, raising intracellular Ca^2+ ^concentration and triggering insulin exocytosis. The factors that modulate or disrupt IP3- mediated Ca^2+ ^signaling exert functional regulatory role in physiological and pathological effects in the pancreatic islets [81]. Thus, we conclude from our results that mGlu-5 receptors have differential expression in brain regions of diabetes and D+I groups as a function of age. Also, the present study reveals the functional regulatory role of glutamate receptors at different concentrations in intracellular calcium release from pancreatic islets *in vitro*. In conclusion, the study elicits immense therapeutic applications of glutamate receptors in memory enhancement and management of degeneration in brain function during diabetes and ageing. Also, the functional role of glutamate receptors in calcium release has clinical significance in insulin secretion and function.

## Competing interests

The authors declare that they have no competing interests.

## Authors' contributions

SB and CSP designed research. SB performed experiments. PKT helped SB in experiments. SB and CSP analyzed the data. SB and CSP wrote the paper. All authors read and approved the final manuscript.
